# A pilot prospective cohort study using experimental quantification of early peripheral nerve regeneration with high-frequency three-dimensional tomographic ultrasound (HFtUS)

**DOI:** 10.1038/s41598-023-42230-x

**Published:** 2023-09-13

**Authors:** Ralph N. A. Murphy, Steven K. Rogers, Waqar Bhatti, Jason K. Wong, Adam J. Reid

**Affiliations:** 1 Blond McIndoe Laboratories, Division of Cell Matrix Biology and Regenerative Medicine, School of Biological Sciences, Faculty of Biology, Medicine and Health, Manchester Academic Health Science Centre, The University of Manchester, Manchester, M13 9PT UK; 2 Department of Plastic Surgery and Burns, Manchester University NHS Foundation Trust, Manchester Academic Health Science Centre, Wythenshawe Hospital, Manchester, M23 9LT UK; 3 Division of Cardiovascular Sciences, Faculty of Biology, Medicine and Health, Manchester University NHS Foundation Trust, Manchester Academic Health Science Centre, School of Medical Sciences, University of Manchester, Oxford Road, Manchester, M13 9WL UK; 4 Manchester Academic Vascular Research and Innovation Centre (MAVRIC), Manchester University NHS Foundation Trust, Wythenshawe Hospital, Manchester, M23 9LT UK; 5 Department of Musculoskeletal Radiology, Manchester University NHS Foundation Trust, Manchester Academic Health Science Centre, Wythenshawe Hospital, Manchester, M23 9LT UK

**Keywords:** Peripheral nervous system, Regeneration and repair in the nervous system

## Abstract

Quantification of peripheral nerve regeneration after injury relies upon subjective outcome measures or electrophysiology assessments requiring fully regenerated neurons. Nerve surgeons and researchers lack objective, quantifiable information on the site of surgical repair and regenerative front. To address this need, we developed a quantifiable, visual, clinically available measure of early peripheral nerve regeneration using high-frequency, three-dimensional, tomographic ultrasound (HFtUS). We conducted a prospective, longitudinal study of adult patients with ulnar and/or median nerve injury of the arm undergoing direct epineurial repair within 5 days of injury. Assessment of morphology, volumetric and 3D grey-scale quantification of cross-sectional views were made at baseline up to 15 months post-surgery. Sensory and motor clinical outcome measures and patient reported outcome measures (PROMs) were recorded. Five participants were recruited to the study. Our data demonstrated grey-scale values (an indication of axonal density) increased in distal stumps within 2–4 months after repair, returning to normal as regeneration completed (4–6 months) with concomitant reduction in intraneural volume as surgical oedema resolved. Two patients with abnormal regeneration were characterized by increased intraneural volume and minimal grey-scale change. HFtUS may quantify early peripheral nerve regeneration offering a window of opportunity for surgical intervention where early abnormal regeneration is detected.

## Introduction

Injury to major peripheral nerves often leads to poor outcomes for patients even after surgical repair. Functional recovery remains uncertain until the regeneration process is presumed to be near complete. Clinical assessment during regeneration relies on relatively primitive clinical examination, the Tinel’s sign, which lacks sensitivity to patients’ subsequent outcome. The gold standard investigation of electro diagnostics is ineffective for several months after treatment due to reliance on end-organ innervation for effective read-out^1^. This electrodiagnostic gap between surgical nerve repair and end-organ reinnervation leaves a window of uncertainty for nerve surgeons and their patients. No imaging techniques exist to quantify or assess the quality of nerve regeneration in early stages after surgical repair, a time when further surgical intervention may be indicated.

The ubiquitous availability of ultrasound (US), relative inexpensiveness and excellent spatial, axial and temporal resolution has led to it becoming the imaging of choice for investigating peripheral nervous system disorders of the upper limb^2^. However there are limited primary studies utilising ultrasound in the assessment of peripheral nerve regeneration in humans. Those studies that exist often use lower frequency probes to identify pathology when clinically indicated^3,4^. In order to explore peripheral nerve regeneration in vivo, investigators have used longitudinal Magnetic Resonance (MR) images and correlated these with other outcome measures^5^. Clinically, MR neurography provides accurate levels of signal intensity to determine location of a lesion and detail regarding surrounding structures but it lacks meaningful intraneural detail. There is a significant trade-off between resolution and field of view which inhibits accurate intra-neural visualisation of long nerves^6^. The next generation of MR imaging with functional MR neurography uses techniques such as Diffusion Tensor Imaging which analyses water diffusion to quantify nerve health. Injured nerves demonstrate disorganised water movement compared to healthy nerves with more organised movement^7^. These image acquisition techniques may improve spatial resolution, providing greater intraneural detail to monitor nerve regeneration however they require a prolonged image-acquisition time (+ /- stronger magnets), which may preclude clinical use^8^. In addition, it is not possible to scan the full length of a nerve in the limb during the image acquisition process^6^.

Ultrasound, in comparison to Magnetic Resonance Imaging (MRI), is more readily available, costs less and provides excellent spatial resolution of nerves in the arm^9,10^. This allows for detailed morphological images to be produced, providing superior intra-neural detail which has the potential to be refined and further interrogated by clinicians and researchers. In comparison to MRI, scan image acquisition takes seconds even when scanning the entire length of upper limb nerves from axilla to hand.

The advent of high-frequency (HF) US transducers (> 15 MHz) and improved software development^11,12^ has enabled identification of more detailed intraneural architecture. It is now possible to differentiate between fascicular structures within peripheral nerves at the level of the wrist^9^ validating a spatial resolution of 0.38 mm^10^. High-frequency, three-dimensional (3D) US (HFtUS) has been used to assess cross-sectional area of healthy median nerves in the forearm^13^; combining this hardware with image recognition algorithm software now provides the opportunity to interrogate morphometric, volumetric and intra-neural 3D grey-scale changes of healthy and injured peripheral nerves. Intra-neural grey-scale, or echogenicity of a peripheral nerve, is influenced by the degree of axonal density^14^ with injured nerves displaying a hypoechoic appearance^15^ which becomes isoechoic when the nerve has regenerated^16^. It remains unclear what ultrasound-based changes occur around the site of peripheral nerve injury (PNI) during the early phases of nerve regeneration and whether these could be used to assess quality of repair and regeneration.

We therefore sought to determine volumetric and intra-neural 3D grey-scale changes, as measured by HFtUS, during nerve regeneration and how they correspond to commonly used clinical outcome measures and patient-reported outcome measures (PROMs), the Disability of Arm, Shoulder and Hand (DASH) scale and the Impact of a Hand Nerve Disorders (iHAND) scale. Specifically, we aimed to qualitatively describe the ultrasound-based 3D morphometric changes and quantify volume and intra-neural 3D grey-scale changes in regenerating nerve segments (proximal stump, repair site and distal stump) compared to contralateral, uninjured control nerves.

## Results

Five participants were recruited to the trial between 3rd September, 2019 and 6th March, 2020 with upper limb median and ulnar nerve injuries directly repaired within 1 week of injury (Table 1). One patient (Participant 001) was lost to follow-up immediately after recruitment, prior to any imaging. Four patients continued longitudinal follow-up. Timing of their follow-up was required to be pragmatic due to the COVID-19 pandemic.

**Table 1:** Patient demographics.

Study I.D	Age	Gender	Race	Hand dominance	Occupation	Injured nerve (forearm)	Mechanism of injury
001	35	F	Bangladeshi	Right	Bank clerk	Left distal 1/3 median nerve	Knife lac
002	33	F	White-British	Right	Care worker	Distal 1/3 right ulnar nerve	Glass lac
003	37	F	Asian/Asian Brit-Indian	Right	Mental health worker	Proximal 1/3 right median nerve	Glass lac
004	27	F	White-British	Right	Cleaner	Distal 1/3 left median nerve (DSH)	Razor blade (DSH)
005	48	M	Asian/Asian Brit-any other asian b/g	Right	Takeaway owner	Right distal 1/3 median and ulnar nerve	Circular saw injury

*b/g* background, *DSH* deliberate self-harm.

### Individual participant follow-up data

#### Participant 002

Participant 002 (33 year old, female, care worker) sustained a glass laceration to the right distal third forearm requiring repair of her ulnar artery and nerve, just proximal to Guyon’s canal, within 24 h of injury under general anaesthetic with tourniquet control.

**Figure 1:**
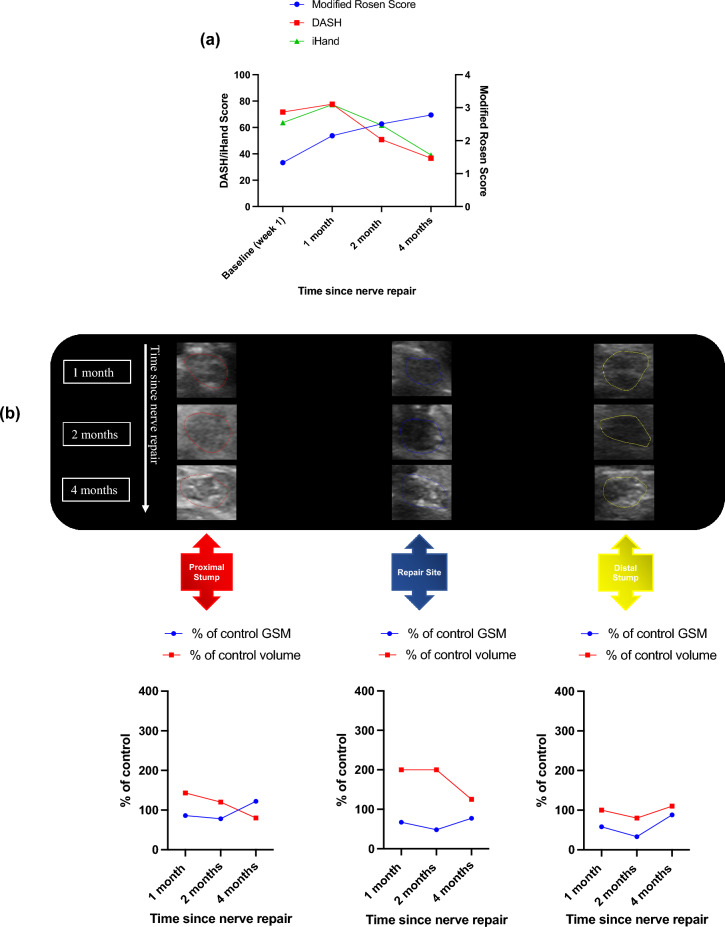
Participant 002 clinical, patient reported and HFtUS outcome data. (**a**) Clinical and patient-reported outcome measures: Disability of Arm Shoulder and Hand (DASH) and Impact of Hand Nerve Disorders (iHand) scores (left-hand y-axis) and Modified Rosen Scores (right-hand y-axis); (**b**) ulnar nerve HFtUS imaging with volumetric and 3D-GSM analysis of participant 002’s injured ulnar nerve at proximal, repair site and distal sections of the nerve and at their corresponding timepoints with the graphical representation of their (%) percentage of control 3D grey-scale median and volumetric (cm^3^) values over these nerve segments. Uniform isoechoic fascicular arrangements can be seen in the proximal stump and repair site at 1–2 months and in the repair site and distal stump at 4 months.

Participant 002 recovered well with good clinical and PROMs by 4 months after surgery. DASH scores improved from 71.7 (week 1) to 36.7 (4 months) and iHand 63.7 to 39.1. Modified Rosen Scores demonstrated an improvement from 1.33 at week 1 to 2.78 at 4 months (Fig. 1a).

Proximal stump 3D-GSM values remained close to 100% of contralateral control nerve values over the time period (86%, 78%, 122%) whilst both repair site and distal stump values initially decreased compared to control at 2 months (48% and 33% respectively) and then increased to 77% and 88% of control values by 4 months as expected with axonal regeneration. The repair site volume was at 200% of the contralateral control from 1 month until it returned to 125% of control at the 4-month visit. Proximal stump volume was 143% of control at 1 month prior to decreasing to 80% of control by 4 months. Distal nerve stumps remained at similar volumes to control at all assessment time points (Fig. 1b).

#### Participant 003

Participant 003 (37 year old, female, mental health worker) suffered a glass laceration to the right proximal 1/3 forearm resulting in transection of the right median, radial and lateral antebrachial cutaneous nerves, brachial artery and biceps muscle.

Participant 003 was managed initially in a major trauma centre undergoing emergency exploration of her right arm wound and brachial artery interposition bypass grafting (using long saphenous vein from right thigh) with prolene suture tagging of the transected nerves and forearm flexor compartment fasciotomy.

Four days later, she underwent direct repair of her nerve injuries using epineurial interrupted suturing under general anaesthetic with tourniquet control.

**Figure 2:**
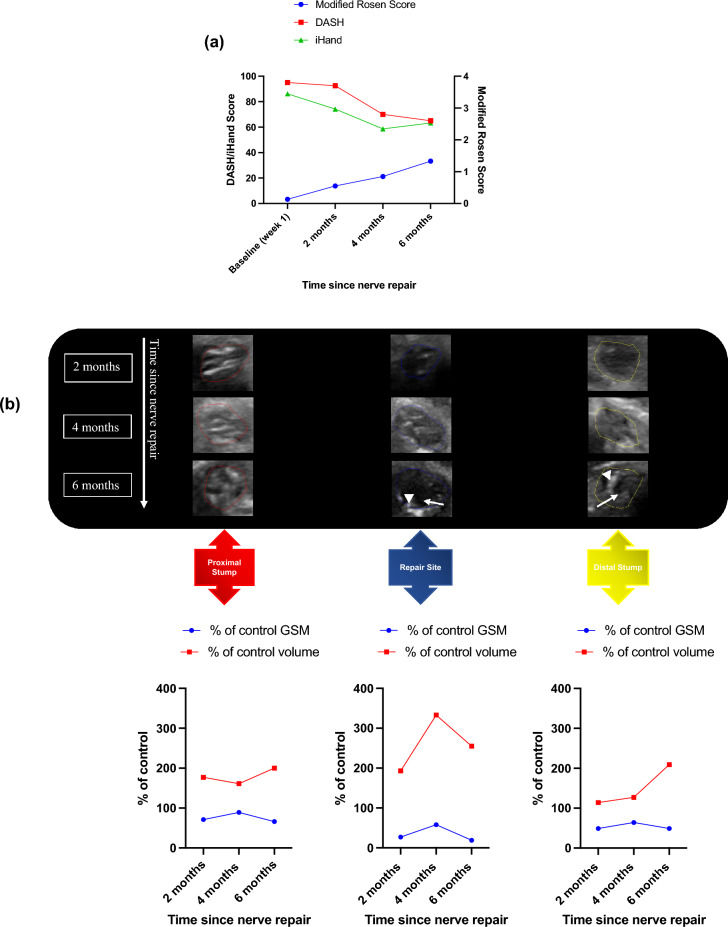
Participant 003 clinical, patient reported and HFtUS outcome data. (**a**) Clinical and patient-reported outcome measures: Disability of Arm Shoulder and Hand (DASH) and Impact of Hand Nerve Disorders (iHand) scores (left-hand y-axis) and Modified Rosen Scores (right-hand y-axis); (**b**) cross-sectional tUS images of participant 003’s injured median nerve at proximal, repair site and distal sections of the nerve and at their corresponding timepoints with the graphical representation of their (%) percentage of control 3D grey-scale median and volumetric (cm^3^) values over these nerve segments. A hypoechoic mass filling the repair site and extending to the distal stump by 6 months represents a neuroma-in-continuity (white arrows). Possible scar tissue band formation which could be contributing to the neuroma formation (white arrow heads).

The patient-reported and clinical outcomes were poor throughout post-operative rehabilitation: there was minimal improvement in PROMs from week 1 to 6 months (DASH 95 to 65 and iHand 86.2 to 63.3); whilst modified Rosen Scores demonstrated little clinical improvement from 0.13 at week 1 to 1.33 at 6 months (Fig. 2a).

The images in Fig. 2b show no 3D-GSM changes between 2 and 6 months after repair: proximal stump 71% of control at 2 months and 66% of control by 6 months; repair site 27% and 19%; and distal stump values of 49% and 49% respectively. Proximal stump volume was 177% of control volume at 2 months and whilst decreasing slightly at 4 months (161%) it remained enlarged at 6 months (200% of control). Repair site volume increased from 193% of control at 2 months to 333% of control at 4 months and by 6 months was still 255% of control volume. Distal stump volume stayed relatively static with a value of 114% of control at 2 months, 127% of control at 4 months and then jumped in volume to 209% of control by 6 months.

**Figure 3:**
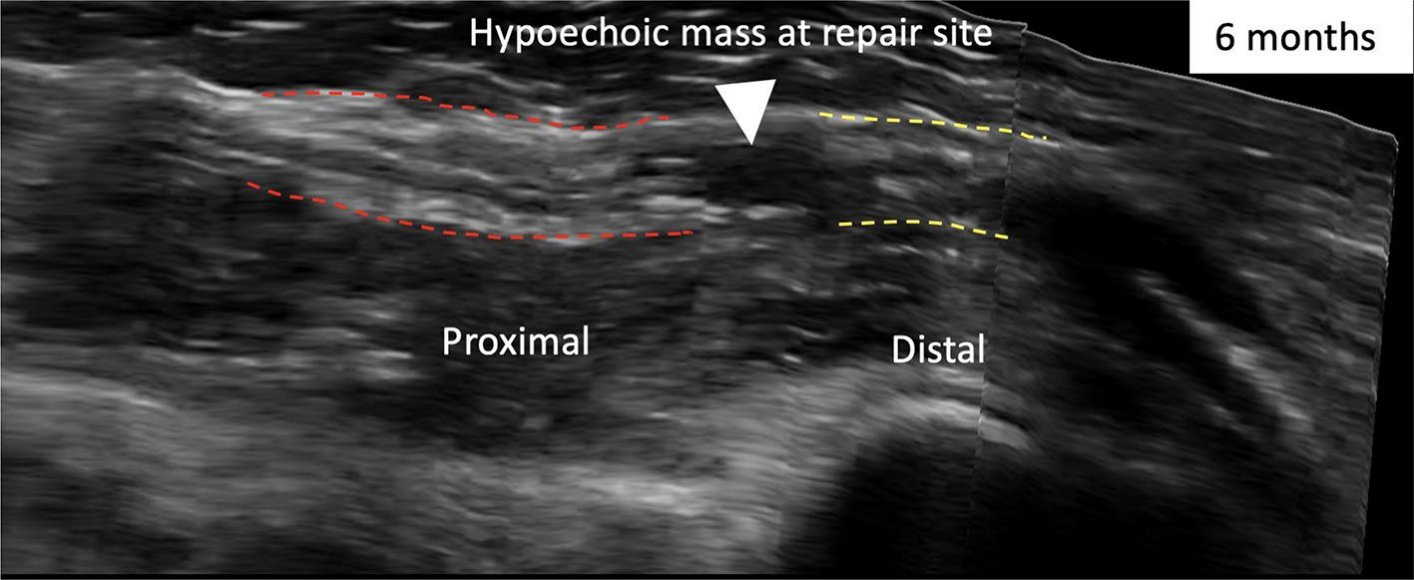
Sagittal-view with 3D reconstruction of participant 003’s injured median nerve at 6 months. There is a hypoechoic (black) mass at the repair site (white arrowhead) and incomplete fascicles from proximal (red-dash lines) to distal stumps (yellow-dash lines). The epineurium around the repair site appears intact and therefore represents a neuroma-in-continuity.

A neuroma-in-continuity had developed during regeneration which was characterised in 3D sagittal views at 6 months post-repair (Fig. 3).

#### Participant 004

Participant 004 (27 years old, female, cleaner) sustained deliberate self-harm injury with a razor blade to the left distal third forearm. Surgery was undertaken the following day to repair the flexor carpi radialis tendon, median nerve and ulnar artery under general anaesthetic with tourniquet control.

**Figure 4:**
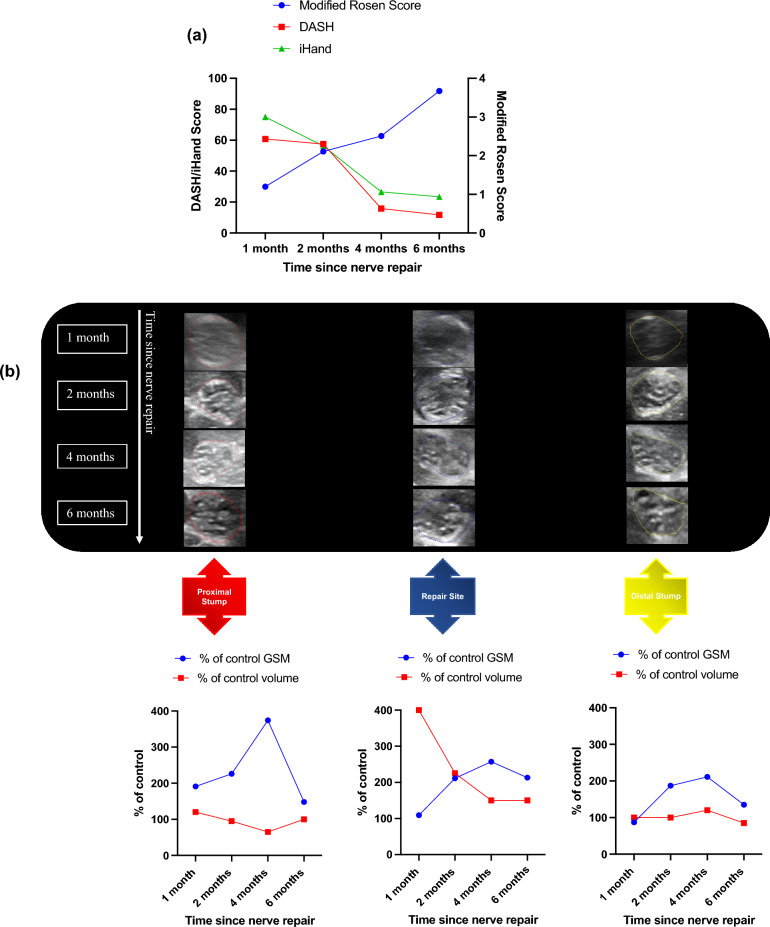
Participant 004 clinical, patient reported and HFtUS outcome data. (**a**) Clinical and patient-reported outcome measures: Disability of Arm Shoulder and Hand (DASH) and Impact of Hand Nerve Disorders (iHand) scores (left-hand y-axis) and Modified Rosen Scores (right-hand y-axis); (**b**) cross-sectional tUS images of participant 004’s injured median nerve at proximal, repair site and distal sections of the nerve and at their corresponding timepoints with the graphical representation of their (%) percentage of control 3D grey-scale median and volumetric (cm^3^) values over these nerve segments. There is a uniform organised fascicular arrangement across all segments. Hyperechoic from 2 to 4 months, becoming isoechoic by 6 months.

Participant 004 had excellent patient-reported and clinical outcomes during post-operative rehabilitation. Both DASH 60.83 to 11.67 and iHand 75 to 23.44 greatly improved from 1-month to 6-months respectively after surgery. Modified Rosen Scores were also much improved from a value of 1.2 at 1 month to 3.67 by 6 months (Fig. 4a).

There is a clear increase in 3D-GSM in all sections of the repaired nerve between 1 and 4 months (Fig. 4b): proximal stump values increased from 191% of control at 1 month to 374% of control by 4 months; repair site values increased from 109% of control at 1 month to 257% of control by 4 months; and distal stump values increased from 87% of control at 1 month to 211% of control by 4 months. 3D-GSM value of all segments then returned towards control by 6 months to be 148% (proximal stump), 213% (repair site) and 135% (distal stump).

Change in volume of nerve was most marked at 1 month in the repair site to 400% of control with proximal and distal stumps remaining close to control volumes. Repair site volume gradually decreased to a value of 150% of control by 6 months whilst proximal and distal stump volumes remained largely unchanged throughout, staying at values close to the contralateral control volume (Fig. 4b).

#### Participant 005

Participant 005 (48 year old, male, takeaway shop owner) sustained a right distal third forearm laceration from an angle grinder. Surgery was undertaken the following day with the median and ulnar nerves, ulnar artery and all tendons (FPL, FCR, FCU, PL and FDS and FDP to all digits) repaired under general anaesthetic with tourniquet control.

##### Median nerve

**Figure 5:**
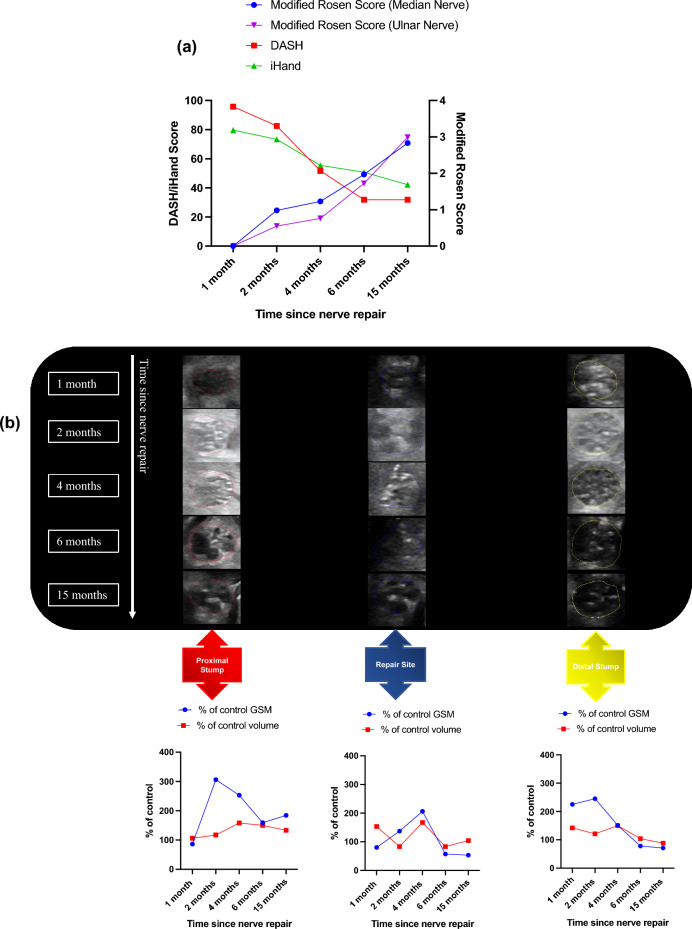

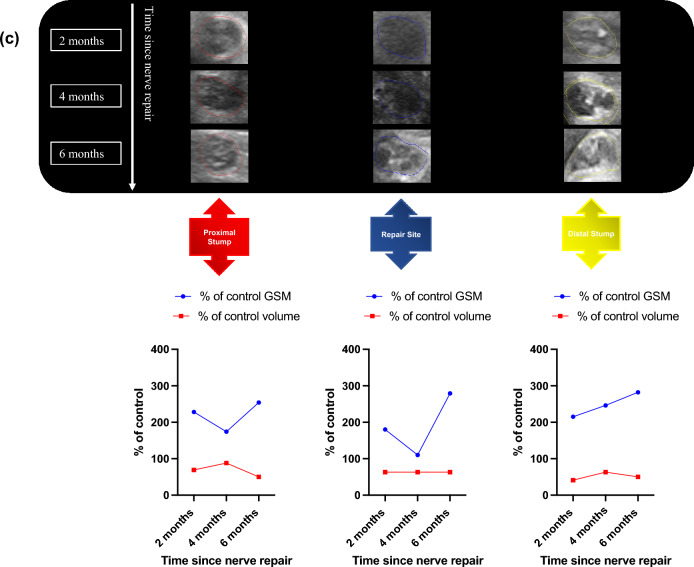
Participant 005 clinical, patient reported and HFtUS outcome data. (**a**) Clinical and patient-reported outcome measures: Disability of Arm Shoulder and Hand (DASH) and Impact of Hand Nerve Disorders (iHand) scores (left-hand y-axis) and Modified Rosen Scores (right-hand y-axis) for both median and ulnar nerves; (**b**) cross-sectional tUS images of participant 005’s injured median nerve at proximal, repair site and distal sections of the nerve and at their corresponding timepoints with the graphical representation of their (%) percentage of control 3D grey-scale median and volumetric (cm^3^) values over these nerve segments. Between 6 and 15 months there is a isoechoic fascicular pattern in the proximal stump and hypoechoic fascicular pattern in the repair site and distal stumps; (**c**) cross-sectional tUS images of participant 005’s injured ulnar nerve at proximal, repair site and distal sections of the nerve and at their corresponding timepoints with the graphical representation of their (%) percentage of control 3D grey-scale median and volumetric (cm^3^) values over these nerve segments. Between 4 and 6 months there is a hypoechoic fascicular pattern in the repair site and distal stumps.

He had good patient-reported and clinical recovery. Both DASH 95.83–31.9 and iHand 79.69–42.19 improved from 1 to 15 months after surgery. Modified Rosen Scores improved from a value of 0 at 1 month to 2.83 by 15 months (Fig. 5a).

The images in Fig. 5b demonstrate large increases in 3D-GSM values from 1 to 2 months after repair in all segments (proximal 86–306%, repair site 80–137% and distal 224–245%). These values began a downward trend from 4 months through to 15 months (proximal 253–184%, repair site 206–53% and distal 151–71%).

Volumes of all segments increased from 1 to 4 months after repair (proximal 106–158%, repair site 153–167% and distal 142–150%) followed by a decrease through to 15 months after repair (proximal 158–113%, repair site 167–104% and distal 150–88%) (Fig. 5b).

##### Ulnar nerve

Modified Rosen Scores improved from a value of 0.55 at 2 months to 1.72 by 6 months (going on to a score of 2.99 by 15 months) (Fig. 5a).

Images in Fig. 5c also show decreases in 3D-GSM values from 2 to 4 months after repair in proximal stump (228–174%) and repair sites (180–110%), whereas there was an increase in 3D-GSM in the distal stump (215–246%). From 4 to 6 months after repair, all segments demonstrated an increase in 3D-GSM values (proximal 174–254%, repair site 110–279% and distal 246–282%).

There was little change in volumes of all segments throughout the observation period.

## Discussion

Ultrasound is readily available, costs significantly less than magnetic resonance imaging (MRI) and provides excellent spatial resolution of nerves in the arm^9,10^. Detailed morphological images can be produced, providing superior intra-neural detail with superb spatial and temporal resolution with the potential to be refined and further interrogated by clinicians and researchers. Compared to MRI, scan image acquisition takes seconds, even when scanning the entire length of upper limb nerves from axilla to hand. Ultrasound is therefore regarded as the initial imaging modality of choice in primary imaging of upper limb nerves.

In this pilot study, we used high-frequency probes (20 mHz) and 3D, tomographic software to obtain detailed neuronal architectural images of the early stages of peripheral nerve regeneration in human participants undergoing direct neurorrhaphy. We analysed the ultrasound-based geometry and morphometry using novel image analysis software (PIUR imaging, Vienna, Austria) to quantify intraneural 3D grey-scale (echogenicity) and volumetric changes during regeneration at the repair site and proximal/distal stumps. These areas were chosen and standardised between participants to allow for direct comparison during regeneration. We scanned contralateral control nerves at baseline to demonstrate uniformity of 3D grey-scale median values along the length of the nerve. We expected regeneration within the areas of examination to be complete prior to 6 months; however, participants had later measurements to explore whether any further changes occurred.

We identified trends in the HFtUS morphology, 3D grey-scale median (echogenicity) and volume measurements that appear to correlate well with clinical and PROMs. Firstly, morphometric analysis allows identification of abnormalities in regeneration and localisation of causative factors, such as participant 003 who developed a neuroma-in-continuity due to intra-neural scarring. These abnormalities appear to correlate strongly with volumetric analysis (participant 003) where areas of abnormal growth, or neuromas-in-continuity, lead to intraneural oedema. Volumetric changes at the repair site also helped confirm whether post-operative oedema had resolved (participant 004).

**Figure 6:**
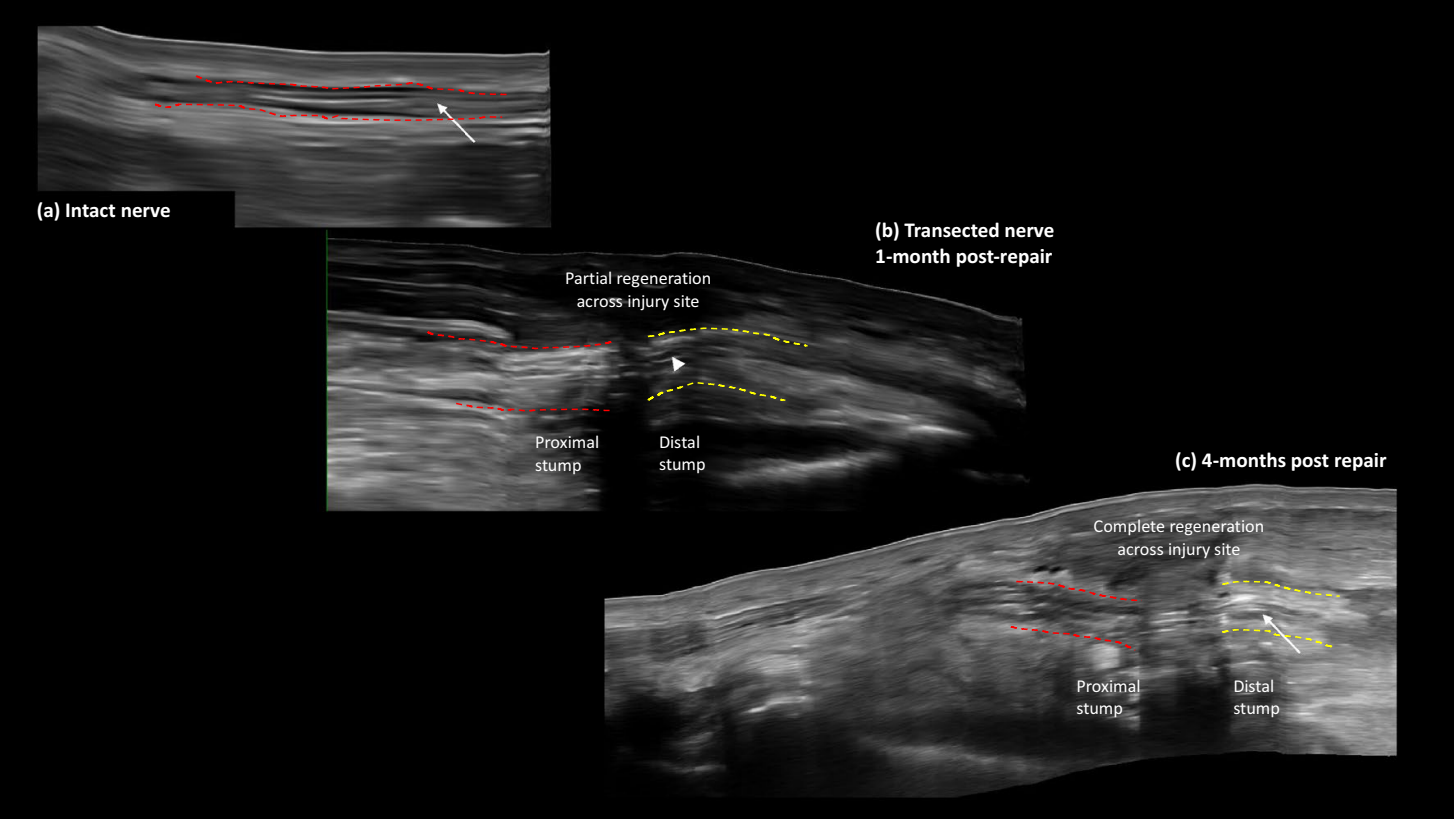
HftUS sagittal views of: (**a**) an intact peripheral nerve with isoechoic nerve fascicles (white arrow); (**b**) a transected peripheral nerve 1-month after repair with hypoechoic nerve fascicles (white arrowhead) in distal stump and (**c**) a transected peripheral nerve 4-months after repair. Distal stump fascicles becoming increasingly isoechoic (white arrow). Red-dashed lines represent epineurium of intact nerve (**a**) and proximal stump in transected nerves (**b**) and (**c**). Yellow-dashed lines represent epineurium of distal stump in transected nerves (**b**) and (**c**).

3D-GSM allows quantification of the degree of echogenicity of intraneural tissues for the first time. Detailed echogenic analysis of specific injured nerve segments in 3D has never previously been described and may enable quantification of nerve regeneration. In vivo studies of peripheral nerve regeneration using MRI has previously demonstrated hyperintense signals distal to the injury which subsequently regress in a proximal to distal direction in keeping with evidence of electrophysiological and histological regeneration^5^. We demonstrated an increase in 3D-GSM from proximal to distal across the repair site before decreasing back to levels of the contralateral control uninjured nerves once regeneration is complete (Fig. 6). This may be explained by the fact that collagen and myelin are more hyperechoic than collagen alone^17^, thus laying down of new myelin as the nerve regenerates presents a more hyperechoic signal (Fig. 6c) than the empty collagen “shell” of a nerve fascicle that has undergone Wallerian degeneration in the distal stump (Fig. 6b). This increase in echogenicity in the distal stump fascicles returns to that of an uninjured nerve when the post-operative intraneural oedema has resolved (Fig. 6a and c).

Overall, participants with good clinical outcome measures (Participants 002, 004 and 005) had HFtUS findings as we might expect with a hyperechoic, uniform fascicular pattern from proximal to distal stump at 1 month after surgical repair returning to an isoechoic fascicular pattern by 6 months. Volume increase at repair sites ranged from 1—4 × the contralateral control, returning towards control levels by 4—6 months; whilst proximal and distal stumps had minimal net volume change. 3D-GSM increased in all areas after repair, the greatest increases occurring in the proximal stump with repair site and distal stump changes increasing sequentially as the nerve regenerated. Between 4 and 6 months after injury 3D-GSM values returned towards control values as nerve regeneration was expected to be complete and as clinical outcome measures plateaued.

In the participant with poor clinical outcome measures (Participant 003), HFtUS findings demonstrated a disorganised fascicular pattern across all segments and by 6 months a neuroma-in-continuity had developed (confirmed by sagittal views). The volume measurements in the proximal stump at 2 months was double the control as opposed to other patients where this had returned to normal. At the repair site, whilst an increase in volume at 2 months was seen in other patients, by 4 months this continued to increase in participant 003 rather than return to normal. 3D-GSM values remained significantly lower than the control throughout the follow-up period in all segments. Of note, all data was examined at the end of the study so clinical decision making was not impacted by the HFtUS scans and subsequent findings; however, there was clear evidence of a difference in volume in the proximal stump at the 2 month and at the repair site at 4 month timepoints which with greater understanding and analysis of this technique may provide the window of opportunity for earlier surgical intervention to mitigate failed regeneration.

These ultrasound-based biomarkers of peripheral nerve regeneration may provide nerve surgeons and researchers with a novel (but affordable and available), early and objective measure of peripheral nerve regeneration in humans. Clinically important changes can be detected in the first 2—4 months after repair which is a critical window of opportunity to intervene and currently lacks sensitive diagnostics. HFtUS is simple in use and does not rely upon on peripheral nerve expertise to capture appropriate images for downstream analysis in specialist software which allows for detailed interrogation of neuronal structural changes in three planes (cross-section/transverse, sagittal and coronal). Equipment, in addition to a standard ultrasound machine, is a laptop and a probe attachment approximately the size of a printer cartridge. Cost is less than MRI and intuitive image analysis can be performed by peripheral nerve surgeons with minimal prior ultrasound training. The first author responsible for image analysis required two clinical half days and subsequent reading to develop the knowledge and skills required.

This pilot study was limited by participant recruitment and irregular follow-up visits due to national pauses in face-to-face clinical research during the coronavirus pandemic. However, it provides a clear structure and basis to inform a future prospective, multicentre, adequately powered clinical investigation to: validate the relationship between these novel ultrasound biomarkers and clinical and PROMs of peripheral nerve regeneration and establish inter-observer validity and reliability of this technique. To broaden the utility, investigations of these biomarkers will be required at prolonged time-points of denervation to replicate the clinical reality of referral into specialist centres, alongside other typical clinical contexts (peripheral nerve compression versus blunt injury). This study was also limited by the lack of available PNI-specific PROMs to compare with novel imaging techniques such as the work presented. The PROMs utilised fail to capture features important to PNI patients in particular in motor function, psychology and well-being which is a major limitation in the field of PNI research. Further PROMs specific to PNI will be required to demonstrate effectiveness of new interventions and validate novel outcome measures of peripheral nerve regeneration^18^. Addition of ultrasound-contrast may further help to explore the neovascularisation processes during regeneration. This combined with 3D grey-scale median analysis on HFtUS may help highlight areas for targeted therapies or interventions that could improve the regenerative process to ensure outcomes are optimised beyond simple repair strategies.

## Methods

We conducted an open, non-randomised, prospective, longitudinal study of HFtUS characteristics in participants who had undergone surgical repair of a major upper limb peripheral nerve. The study was purely observational and no change to standardised treatment was made. The UK Health Research Authority (HRA) provided ethical permission for the clinical investigation (reference: 18/EM/0426) and reporting follows the Strengthening the Reporting of Observational Studies in Epidemiology recommendations.

Participants were recruited in the Department of Plastic Surgery at Wythenshawe Hospital, Manchester University NHS Foundation Trust. All adults aged 18–80 years old with an ulnar and/or median nerve injury of the upper limb who received direct, epineurial repair within 5 days of injury and had capacity to consent were invited to take part between 3rd September, 2019 and 6th March, 2020. Patient age, gender, ethnic background, hand dominance, occupation, injured nerve, mechanism of injury, significant past medical history and smoking status was recorded. All patients received a standard care hand therapy protocol to protect the nerve repair, early motion (depending on concomitant injuries), oedema control and rehabilitation.

A baseline HFtUS was performed for each patient within first 4 weeks after injury followed by further HFtUS scans at each subsequent follow-up appointment to assess intraneural volume and 3D grey-scale median (3D-GSM) values across proximal, repair site and distal stump segments. At baseline, contralateral, uninjured upper limb nerves were also scanned to obtain a control measurement of intraneural volume and 3D-GSM values to compare contralateral changes as the nerve regenerated. HFtUS techniques are described in greater detail in Supplementary Material 1. Concomitant clinical sensory and motor testing was performed and PROMs completed at each follow-up visit (Supplementary Material 2).

### Ethical approval

The UK Health Research Authority (HRA) provided ethical permission for the clinical investigation (reference: 18/EM/0426), with all research performed in accordance with the Declaration of Helsinki. Informed consent was obtained from all participants.

### Supplementary Information

Supplementary Information 1.

Supplementary Information 2.

## Data Availability

All data generated or analysed during this study are included in this published article (and its supplementary information files).

## References

[1] Dorfman LJ (1990). Quantitative clinical electrophysiology in the evaluation of nerve injury and regeneration. Muscle Nerve.

[2] Ohana M (2014). Current and future imaging of the peripheral nervous system. Diagn. Interv. Imaging.

[3] Peer, S., Bodner, G. *High-Resolution Sonography of the Peripheral Nervous System* (Reptile Medicine and Surgery) 471–489 (2006).

[4] Wijntjes J, Borchert A, van Alfen N (2021). Nerve ultrasound in traumatic and iatrogenic peripheral nerve injury. Diagnostics.

[5] Bendszus M, Wessig C, Solymosi L, Reiners K, Koltzenburg M (2004). MRI of peripheral nerve degeneration and regeneration: Correlation with electrophysiology and histology. Exp. Neurol..

[6] Rangavajla, G., Mokarram, N., Masoodzadehgan, N., Pai, S. B., & Bellamkonda, R. V. Noninvasive imaging of peripheral nerves. in *Cells Tissues Organs* vol. 200, ed: NIH Public Access, 69–77 (2014). 10.1159/000369451 PMC4494672 25766202

[7] Noguerol TM, Barousse R, Cabrera MG, Socolovsky M, Bencardino JT, Luna A (2019). Functional MR neurography in evaluation of peripheral nerve trauma and postsurgical assessment. Radiographics.

[8] Lehmann HC, Zhang J, Mori S, Sheikh KA (2010). Diffusion tensor imaging to assess axonal regeneration in peripheral nerves. Exp. Neurol..

[9] Suk JI, Walker FO, Cartwright MS (2013). Ultrasonography of peripheral nerves. Curr. Neurol. Neurosci. Rep..

[10] Brill NA, Tyler DJ (2017). Quantification of human upper extremity nerves and fascicular anatomy. Muscle Nerve.

[11] Fornage BD (1988). Peripheral nerves of the extremities: imaging with US. Radiology.

[12] Stuart RM, Koh ESC, Breidahl WH (2004). Sonography of peripheral nerve pathology. Am. J. Roentgenol..

[13] Pelz JO, Busch M, Weinreich A, Saur D, Weise D (2004). Evaluation of freehand high-resolution 3-dimensional ultrasound of the median nerve. Muscle Nerve.

[14] Powles, A. E. J., Martin, D. J., Wells, I. T. P., & Goodwin, C. R. *Physics of Ultrasound*, Fifth Edit ed. (Anaesthesia and Intensive Care Medicine) 202–205 (Elsevier Inc., 2018).

[15] Mayans D, Cartwright MS, Walker FO (2012). Neuromuscular ultrasonography: Quantifying muscle and nerve measurements. Phys. Med. Rehabil. Clin. N. Am..

[16] Hudson JA, Steiss JE, Braund KG, Totvio-Kinnucan M (1996). Ultrasonography of peripheral nerves during Wallerian degeneration and regeneration following transection. Vet. Radiol. Ultrasound.

[17] Byra M (2019). Quantitative ultrasound and B-mode image texture features correlate with collagen and myelin content in human ulnar nerve fascicles. Ultrasound Med. Biol..

[18] Murphy RNA, Elsayed H, Singh S, Dumville J, Wong JKF, Reid AJ (2021). A Quantitative Systematic Review of Clinical Outcome Measure Use in Peripheral Nerve Injury of the Upper Limb. Neurosurgery.

