# Supplementary material for: A pilot prospective cohort study using experimental quantification of early peripheral nerve regeneration with high-frequency three-dimensional tomographic ultrasound (HFtUS)
Source: Sci Rep. 2023 Sep 13;13:15175. doi: 10.1038/s41598-023-42230-x (PMC10499886; doi:10.1038/s41598-023-42230-x)
Supplement: Supplementary file 1 — Supplementary Information 1. [file 41598_2023_42230_MOESM1_ESM.docx]

**Supplementary Material 1**

HFtUS

Participants underwent HFtUS in the Vascular Studies Unit, performed by one of the authors, a Clinical Scientist (SR) with expertise and training in 2D and 3D vascular US. Scans were produced using a linear 20 MHz transducer, a Resona 7 high-resolution US scanner (Mindray, Shenzhen, China) and a standard nerve imaging pre-set. This was connected to a PIUR tUS system (PIUR Imaging, Vienna, Austria). The transducer had electromagnetic sensors attached and tUS images were captured from the Resona via video capture. Individual 2D ultrasound frames were extracted and compiled using the PIUR tUS system to create a 3D image reconstruction in near real-time. All scans were performed using a nerve pre-set compression curve in B-Mode with standardised settings of dynamic range = 110, persistence = 2 and a starting gain of 48 that produced a frame rate of at least 62 frames per second. Sound speed compensation was individually set to each patient for dynamic focusing on reception. Time gain compensation was set diagonally and not adjusted. Minimal gain adjustments were made on each participant to optimise the images. The 20MHz probe frequency was chosen as an optimal balance between neural architectural detail and technical difficulty in tracking nerves without continual adjustments when obtaining scan images as this degrades 3D image quality

Patients were positioned in the most comfortable position depending on their injury pattern and their arms were placed in an extended position at the elbow and supine hand and wrist position. The original injury site was marked with further marks made at 5 cm proximal and distal to this wound overlying the position of the relevant nerve. Scans were performed from a proximal to distal direction in one continuous smooth motion incorporating a similar length of nerve in each scan and at each visit producing a fused series of tUS scans. During initial baseline scans, where some wounds were not fully healed, aseptic non-touch procedures were employed, whereby the wounds were exposed from overlying dressings, a sterile transparent primary dressing was applied (Tegaderm™, 3M™ Minnesota, USA) and a sterile probe cover applied to allow application of sterile ultrasound gel and probe contact to patients’ arms without contaminating the wound. Fresh dressings were then applied after the scan. At baseline, contralateral uninjured nerves, and injured nerves, were scanned in order to provide an internal control grey-scale value and volume measurement for future injured nerve scans.

Image analysis was performed using PIUR imaging (Vienna, Austria) 3D, tomographic ultrasound system (tUS) software (version 2.1 #15) [1]. A plastic surgeon trained in ultrasound image analysis performed the analysis of the regenerating nerve images after all images were obtained. Analysis was undertaken by the first author following training by a Professor of Musculoskeletal Imaging. Fused tUS scans were used to measure segmental nerve volume using a standard technique to measure digital artery volumes [2] and were presented in centimetres-cubed (cm^3^). Peripheral nerve analysis software developed by PIUR imaging (Vienna, Austria) was used to measure the intraneural three-dimensional-grey scale median (3D-GSM) value in each segment. The 3D-GSM is calculated, taking all voxels (volume and pixel) inside the segmentation into account and ranges from 0 (black) to 255 (white) [3] . A standardised distance of 20mm from repair site was used for proximal and distal stump analysis, where possible, with a variable length of repair site reported based on its clear morphological appearance during scanning. The overlying cutaneous scar served as a reference point to locate the repair site. Each analysis of volume and 3D-GSM was repeated three times with the third and final result utilised. Results were presented as a percentage of the contralateral control 3D-GSM. Contralateral, un-injured control nerves scanned at the baseline visit, at the same anatomical location as the injured nerve, were segmentally (10mm) analysed to establish the volume and 3D-GSM variability along their length. A mean control volume (cm^3^) and 3D-GSM was subsequently calculated and results from the injured nerve were presented as a percentage of the control values.

**References:**

[1] D. B. Downey, A. Fenster, and J. C. Williams, "Clinical utility of three-dimensional US," *Radiographics,* vol. 20, no. 2, pp. 559-571, 2000, doi: 10.1148/radiographics.20.2.g00mc19559.

[2] M. Hughes *et al.*, "Imaging digital arteries in systemic sclerosis by tomographic 3-dimensional ultrasound," *Rheumatology International,* vol. 41, no. 6, pp. 1089-1096, 2021, doi: 10.1007/s00296-020-04675-5.

[3] I. B. Casella, R. B. Fukushima, A. B. d. A. Marques, M. V. M. Cury, and C. Presti, "Comparison between a new computer program and the reference software for gray-scale median analysis of atherosclerotic carotid plaques," *Journal of Clinical Ultrasound,* vol. 43, no. 3, pp. 194-198, 2015, doi: 10.1002/jcu.22178.
