# Supplementary material for: A pilot prospective cohort study using experimental quantification of early peripheral nerve regeneration with high-frequency three-dimensional tomographic ultrasound (HFtUS)
Source: Sci Rep. 2023 Sep 13;13:15175. doi: 10.1038/s41598-023-42230-x (PMC10499886; doi:10.1038/s41598-023-42230-x)
Supplement: Supplementary file 2 — Supplementary Information 2. [file 41598_2023_42230_MOESM2_ESM.docx]

**Supplementary Material 2**

Clinical Testing

*Sensory Testing;* Three sensory tests were utilised to measure sensibility outcomes including: the Weinstein Enhanced Sensory Test (WEST) to assess single-point pressure threshold and the integrity of the afferent fibre populations, the static two-point discrimination test (S2PD) to assess sensory discrimination and locognosia testing to assess functional sensibility of the injured nerves. Testing was performed by the primary author (RM) competent in using the described sensory testing techniques.

All tests were performed in a quiet room with the patient sitting opposite the examiner with their arms resting on a pillow, elbows at ~45-90 degrees of flexion and semi-supination of the wrists. This position was chosen as the most comfortable position for most patients to sit through the sensory testing without movement of the hands. The hands were then placed through a JAMAR sensory testing shield (Performance Health, IL, USA) to blind the patient to the stimulus during the examination.

The WEST monofilament tool (Fabrication Enterprises Inc., NY, USA) developed by Sidney Weinstein [1] was used for pressure threshold testing. This tool includes five standard filaments which apply a standard calibrated force of 0.07g, 0.2g, 2.0g, 4.0g and 200g. For the purposes of this study a corresponding integer from 5 (0.07g) to 1 (200g) represented the incremental monofilaments with 0 representing an inability to detect any force. A random monofilament test was employed whereby each filament was applied in random order to the test sites with the lowest detectable stimulus recorded in terms of the corresponding integer.

The S2PD test was performed incrementally using a blunt-tipped discriminator (Baseline DISCRIM-A-GON, Fabrication Enterprises Inc., NY, USA) as described by Mackinnon and Dellon [2] starting with the smallest width and just sufficient pressure for the subject to detect the stimulus. Each pin set distance was performed 10 times and a score of 7 positive responses out of 10 was deemed a positive response.

The locognosia test was performed as described by Jerosch-Herold et al. JBJS 2006 [3]. A diagram of the hand with a superimposed grid of zones, numbered is presented to the patient. The patient is asked to identify the zone where a suprathreshold stimulus has been perceived. The stimulus is delivered using a WEST monofilament (Fabrication Enterprises Inc., NY, USA), which upon contact with the skin bends, providing a repeatable peak force of 200g.

*Motor Testing;* two motor tests were employed, with the patient in the same position. Manual muscle testing utilising the Medical Research Council scale [4] was performed as described except with the examiner utilising the same tendon/muscle group to oppose that being tested in the patient. Isometric grip force was measured using a JAMAR Smart Hand Dynamometer (Performance Health, IL, USA) with a mean of 3 trials in the second position using the uninjured followed by the injured side. Scores of both the manual muscle testing and isometric grip force were recorded as per the Rosen Score [5].

A modified version of the Rosen Scoring system [5] was utilised in order to generate a combined sensory and motor score. The STI test and Sollerman test were removed on advice of the hand therapy department due to lack of use in clinical practice. They were replaced with locognosia testing as previously described above with the injured score represented as a quotient of the contralateral control score with a maximum score of 5 achievable if the injured scoring matched the control scoring. No change was made to the Semmes-Weinstein monofilament, S2PD or motor score reporting whilst cold intolerance and hyperaesthesia testing were excluded as they were also not routinely performed in clinical practice.

*Patient-Reported Outcome Measures (PROMs)*

Two patient-reported outcome measures were utilised. The Disabilities of the Arm, Shoulder and Hand (DASH) measure, which is a 30-item (scored 1-5) self-reported questionnaire reported as a percentage whereby the sum of responses is divided by number of responses minus 1 and multiplied by 25 (Institute for Work and Health, 2006). And the Impact of Hand Nerve Disorders (I-HaND) scale V2 which is a 32-item (scored 1-5) self-reported questionnaire, reported as a percentage as described for the DASH score [6].

**References**

[1] S. Weinstein, "Fifty years of somatosensory research: From the Semmes-Weinstein Monofilaments to the Weinstein Enhanced Sensory Test," *Journal of Hand Therapy,* vol. 6, no. 1, pp. 11-22, 1993/1// 1993, doi: 10.1016/S0894-1130(12)80176-1.

[2] S. E. Mackinnon and A. L. Dellon, "Two-point discrimination tester," *Journal of Hand Surgery,* 1985, doi: 10.1016/S0363-5023(85)80173-8.

[3] C. Jerosch-Herold, "The reliability and validity of the locognosia test after injuries to peripheral nerves in the hand," *Journal of Bone and Joint Surgery - British Volume,* vol. 88-B, no. 8, pp. 1048-1052, 2006/8// 2006, doi: 10.1302/0301-620X.88B8.17444.

[4] B. G. Riddoch *et al.*, "Aids to the examination of the peripheral nervous system," *Medical Research Council,* 1976.

[5] B. Roseén and G. Lundborg, "A model instrument for the documentation of outcome after nerve repair," *Journal of Hand Surgery,* vol. 25, no. 3, pp. 535-543, 2000/5// 2000, doi: 10.1053/jhsu.2000.6458.

[6] M. Ashwood, C. Jerosch-Herold, and L. Shepstone, "Development and validation of a new patient-reported outcome measure for peripheral nerve disorders of the hand, the I-HaND© Scale," *Journal of Hand Surgery (European Volume),* pp. 175319341878055-175319341878055, 2018/6// 2018, doi: 10.1177/1753193418780554.
